# Double Heterotopic Pregnancy: Challenges in Diagnosis and Management

**DOI:** 10.7759/cureus.107557

**Published:** 2026-04-22

**Authors:** Elie Hobeika, Dalia Malaeb, Dina Chamsy, Labib Ghulmiyyah, Nabil El Khoury, Anwar Nassar

**Affiliations:** 1 Obstetrics and Gynecology, American University of Beirut Medical Center, Beirut, LBN; 2 Obstetrics and Gynecology, Kanad Hospital, Abu Dhabi, ARE

**Keywords:** bilateral tubal, case report, diagnosis, heterotopic pregnancy, management

## Abstract

Heterotopic pregnancy is a rare complication; however, its incidence is increasing. Double heterotopic pregnancy is an even rarer and potentially life-threatening condition. It refers to a situation in which a pregnant woman has at least one intrauterine pregnancy along with two ectopic pregnancies.

We report a case of double heterotopic pregnancy, comprising one intrauterine pregnancy and two ectopic pregnancies, one in each fallopian tube, in a 27-year-old woman. The diagnosis was confirmed during laparoscopic management of a presumed single heterotopic pregnancy, with intraoperative suspicion of a contralateral ectopic pregnancy. Conception occurred via intrauterine insemination following ovulation induction. The pregnancy subsequently resulted in a full-term vaginal delivery of a healthy male infant.

Atypical forms of heterotopic pregnancy involving more than one ectopic gestation are extremely rare. Heterotopic pregnancy is life-threatening and poses significant challenges in both diagnosis and management, particularly in cases of double heterotopic pregnancy. An early and accurate diagnosis, along with timely management, whether medical or surgical, is key to a good prognosis and is considered life-saving for both the mother and the intrauterine pregnancy.

## Introduction

Heterotopic pregnancy is a form of multiple pregnancy. It is defined as the simultaneous presence of an intrauterine pregnancy and an extrauterine ectopic pregnancy. It was first reported in 1708 by DuVernay as an incidentally discovered case upon autopsy of a patient who died due to a ruptured ectopic pregnancy [1]. Today, heterotopic pregnancy is considered a rare complication of spontaneous pregnancy, with an estimated incidence of 1/30000 [2]. However, in the context of ovulation induction, the incidence of heterotopic pregnancy is as high as 1/900 when using clomiphene citrate and 1/100 when utilizing assisted reproductive technologies (ART) [3,4]. Double heterotopic pregnancy, on the other hand, is extremely rare, with very few reported cases in the literature [2]. Hence, the ability of a clinician to diagnose and treat heterotopic pregnancy in a timely manner is a very challenging task that must take into consideration the intention to preserve the intrauterine pregnancy [3].

We present a case of bilateral tubal ectopic pregnancies with a singleton intrauterine pregnancy, describing its management as well as the obstetrical outcomes. This case study aims to present a rare case of bilateral heterotopic pregnancy with successful preservation of the intrauterine pregnancy.

## Case presentation

**Figure 1:**
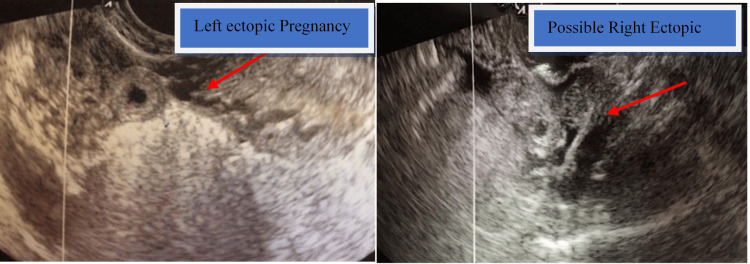
Left and possible right ectopic pregnancies on ultrasound

This is a case of a 27-year-old gravida 1 para 0 (G1P0) patient with polycystic ovarian syndrome and primary infertility. She conceived following ovulation induction with clomiphene citrate and human menopausal gonadotropins, with subsequent intrauterine insemination (IUI). Her workup included normal anti-Müllerian hormone levels, prolactin, thyroid-stimulating hormone (TSH), follicle-stimulating hormone (FSH), and estradiol. A hysterosalpingogram showed a normal uterine cavity and bilaterally patent tubes. Semen analysis demonstrated an abnormal sperm count with decreased motility. The patient had failed to conceive following ovulation induction with timed intercourse and one cycle of in vitro fertilization. This unsuccessful IVF cycle was performed six months prior to the current cycle. The serum beta human chorionic gonadotropin (β-hCG) level at two weeks and two days of gestation was 608 mIU/mL and increased to 1,310 mIU/mL over 48 hours. She presented for her first antenatal visit at six weeks of gestation with lower abdominal discomfort (pain score of 5/10). On bedside ultrasound, a left ectopic pregnancy along with a viable intrauterine pregnancy with positive fetal cardiac activity was identified. In addition, a cystic lesion in the right adnexa measuring 1.2 cm, with surrounding fluid and suspicious for a contralateral ectopic pregnancy, was noted (Figure 1).

**Figure 2:**
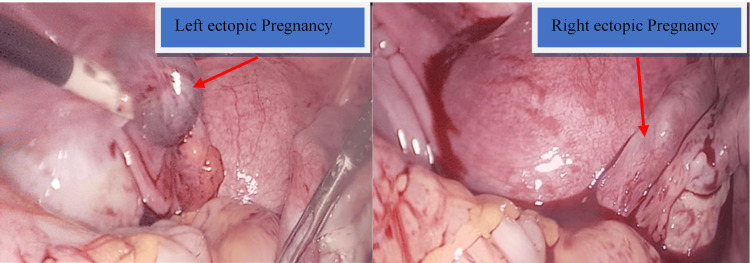
Left and right ectopic pregnancies on laparoscopy

Given the initial findings, the patient was scheduled for diagnostic laparoscopy for the evacuation of the left ectopic pregnancy and evaluation of a possible second right ectopic pregnancy (Figure 2), after discussing the treatment options with her. Intraoperatively, a bilateral heterotopic pregnancy was diagnosed. She subsequently underwent a left-sided salpingostomy with evacuation of the ectopic pregnancy and preservation of the tube. However, the right tube was found to be damaged and could not be salvaged; hence, a right-sided salpingectomy was performed. To preserve the tubes for future conception, salpingostomy is the preferred method over salpingectomy unless the tube is damaged (e.g., ruptured, recurrent ectopic, or dilated more than 5 cm). Thus, the left tube, which was not damaged, was managed with salpingostomy to preserve it, whereas the right tube, which was markedly dilated, was excised.

The patient had a smooth post-operative course, with a positive fetal heart rate of the intrauterine pregnancy, and, subsequently, she was discharged home 12 hours following the procedure. Her antenatal course was also uneventful, as she delivered vaginally at 40 weeks of gestation and was discharged home with the baby in a stable condition.

## Discussion

The above case represents a very rare clinical presentation of a successfully treated bilateral heterotopic pregnancy in which the intrauterine pregnancy was carried to term and delivered without complications. The location of the ectopic gestations bilaterally was tubal, which is where most ectopic pregnancies typically develop. Moreover, while the chance of having a heterotopic pregnancy is exceedingly rare, our patient underwent ovulation induction with clomiphene citrate and human menopausal gonadotropins, which increased the probability of such a rare event to around 1/900 for a single heterotopic pregnancy [3]. Our review of the literature shows that such a rare clinical presentation can be attributed to the use of ART in the absence of typical risk factors for ectopic gestation [5].

The clinical presentation of heterotopic pregnancy is variable and may be misleading at times, especially in the context of a visualized intrauterine gestational sac [4]. The presentation can vary from vaginal bleeding and abdominal pain to gastrointestinal disturbances, or even hypovolemic shock when rupture of the ectopic pregnancy is present at presentation [3]. Moreover, up to 50% of cases of heterotopic pregnancy may be asymptomatic [3]. The ultrasonographic features of heterotopic pregnancy may be confused with physiological ultrasound variants in pregnancy, such as ovarian enlargement (especially in the context of ART) and hemorrhagic corpus luteum cysts [6]. As ART increases the likelihood of heterotopic pregnancy, early ultrasound is an appropriate tool for detecting abnormalities early in pregnancy.

This case highlights the challenging nature of establishing a diagnosis of heterotopic pregnancy, given the normal rise in serum β-hCG levels in the context of a developing intrauterine gestation. The diagnosis of a double heterotopic pregnancy is even more challenging. In our case, ultrasonographic evaluation at six weeks of gestation provided the initial insight into the presence of a left tubal pregnancy coexisting with a viable intrauterine gestation, although there was suspicion of a contralateral ectopic pregnancy. The diagnosis of bilateral heterotopic gestation was confirmed only in the operating room under direct visualization of the tubes by laparoscopy.

Bilateral tubal pregnancy along with a coexisting intrauterine gestation is exceedingly rare, with few reported cases worldwide, mainly following ART and ovulation induction. The rate of live births in bilateral heterotopic pregnancy is similar to that of unilateral heterotopic pregnancy, approaching 60% in the available literature [7]. In the majority of reported cases of bilateral heterotopic pregnancy, laparoscopy was performed. One case was managed expectantly without the need for surgery or medication. It is noteworthy that while in some cases laparoscopic salpingectomy was performed bilaterally, in other cases salpingostomy was performed on one side. We systematically reviewed the literature and identified 18 cases similar to ours. According to Table 1, in a total of 10 reported cases, including ours, the intrauterine pregnancy was carried to term.

**Table 1:** Cases of double heterotopic pregnancies reported in the literature Cases were ordered chronologically. GA: gestational age; ART: assisted reproductive techniques; G: gravida; P: para; IVF: in vitro fertilization; D&C: dilation and curettage; GIFT: gamete intra-fallopian transfer; ICSI: intracytoplasmic sperm injection; IUI: intrauterine insemination

Author	Age	Parity	GA	ART used	No. of transferred embryos	No. of intrauterine sacs	Intervention	Outcome
Hanf et al. [8]	30	G1P0	7	IVF	5	1	Bilateral laparoscopic salpingectomy	Term cesarean delivery
Dietz et al. [9]	35	G2P2	6	Gonadotropins	-	3	Laparoscopic salpingectomy and salpingo-oophorectomy	Miscarriage
Jonler et al. [10]	31	G0P0	6	IVF	-	1	D&C	Abortion
Fujii et al. [11]	23	G1P0	9	Gonadotropins	-	4	Bilateral salpingectomy	Cesarean delivery of twin babies
Wang et al. [12]	29	G0P0	4	GIFT	3	2	Expectant management	Term cesarean delivery
Pan et al. [13]	38	G3P1	7	IVF	3	1	Bilateral salpingectomy	Term vaginal delivery
Hoopmann et al. [2]	39	G0P0	6	ICSI	3	1	Bilateral laparoscopic salpingostomy D&C	Miscarriage
Bettocchi et al. [14]	28	G2P0	7	Gonadotropins	-	5	Laparoscopic salpingectomy and salpingostomy	Term twin cesarean delivery
Nikolic et al. [15]	32	G2P1	10	None	-	1	Bilateral salpingectomy	Term vaginal delivery
Jeong et al. [5]	34	G3P2	8	Clomiphene	-	1	Laparoscopic bilateral salpingectomy and D&C	Miscarriage
Fisher et al. [4]	30	G2P1	11	None	-	-	Operative laparoscopy, evacuation of hemoperitoneum, and bilateral salpingectomy	Multifetal reduction at 13 weeks’ gestation to twins’ delivery
Fukuda et al. [7]	32	G1P0	10	Clomiphene and IUI	-	1	Lap salpingectomy and salpingostomy	Term vaginal delivery
Amine et al. [16]	33	G8P3	9	None	-	1	Bilateral retrograde salpingectomy	Uneventful recovery
Buca et al. [6]	37	-	3	ICSI	3	2	Bilateral salpingectomy	Miscarriage
Mustafa et al. [17]	37	G2P0	8	ICSI	2	2	Bilateral salpingectomy	Miscarriage
Ragam et al. [3]	22	G1P0	9	Ovulation induction with Letrozole	-	1	Bilateral salpingectomy	Term cesarean delivery
Shi et al. [18]	36	G2P0	7	Ovulation induction and IUI	-	1	Bilateral salpingectomy	Term vaginal delivery
Parmar et al. [19]	27	-	6	None	-	1	Bilateral salpingectomy with right ovarian cystectomy	Uneventful recovery
Our case	27	G1P0	6	Clomiphene and gonadotropins	-	1	Laparoscopic salpingectomy and salpingostomy	Term vaginal delivery

The management of heterotopic pregnancy mainly focuses on removing the ectopic component while attempting to preserve the intrauterine gestation. The prognosis of an intrauterine pregnancy in the context of heterotopic gestation is as high as 70% for a successful outcome, according to the current literature [20]. Medical and surgical options exist for this purpose, with varying rates of success and specific risks and side effects. Surgical management is most often performed, accounting for up to 80% of cases in the literature, using laparoscopic or laparotomy techniques [20]. In cases where the ectopic gestation in a heterotopic pregnancy is located in the ampullary or isthmic portion of the tube, salpingectomy is often performed. Medical treatment, including local methotrexate or potassium chloride (KCl) injection into the ectopic sac, suction aspiration, or a combination of these modalities, has also been used, especially in situations where the heterotopic pregnancy is cornual, interstitial, or cervical [20].

The management of our case involved laparoscopic salpingectomy on one side and salpingostomy on the other. This approach to the treatment of bilateral heterotopic pregnancy has been previously reported in two other cases in the literature, both of which were associated with successful continuation of the intrauterine pregnancy to term [7,14].

## Conclusions

Bilateral heterotopic pregnancy is a rare complication that is mainly associated with the use of assisted reproductive techniques in most reported cases in the literature (Table 1). One method to reduce the risk of this condition while using assisted reproduction is to remain attentive to underlying risk factors, including pelvic inflammatory disease, endometriosis, a history of previous ectopic pregnancy or tubal surgery, controlled ovarian hyperstimulation, assisted hatching, and the stage of embryo transfer. Our case underscores the challenging nature of diagnosing heterotopic pregnancy, given the normal increase in serum β-hCG levels in the presence of a developing intrauterine gestation. The ability to promptly diagnose and treat this entity is of utmost importance to ensure patient safety while preserving the desired intrauterine gestation and future fertility. Our case contributes to the limited literature on this topic and may therefore help guide physicians in adequately counseling and managing such a rare condition.
